# Number of recurrences is significantly associated with the post-acute pancreatitis diabetes mellitus in a population with hypertriglyceridemic acute pancreatitis

**DOI:** 10.1186/s12944-023-01840-0

**Published:** 2023-06-29

**Authors:** Xiamin Tu, Qingxie Liu, Lin Chen, Jie Li, Xiaoping Yu, Xiuping Jiao, Ningzhi Wang, Lianghao Hu, Yuan Yuan, Weijuan Gong, Yanbing Ding, Xiaolei Shi, Weiming Xiao, Guotao Lu

**Affiliations:** 1 Pancreatic Center of Gastroenterology, Affiliated Hospital of Yangzhou University, No.368, Hanjiang Middle Road, Yangzhou, 225100 Jiangsu Province China; 2 Yangzhou Key Laboratory of Pancreas in Jiangsu Province, Yangzhou, 225000 Jiangsu China; 3 Health Management Centre, Yangzhou University Affiliated Hospital, Yangzhou, 225000 Jiangsu China; 4 Department of Gastroenterology, Changhai Hospital of the Second Military Medical University, Shanghai, 200433 China; 5 Department of Nursing, Yangzhou University Affiliated Hospital, Yangzhou, 225000 Jiangsu China; 6 College of Medicine, Yangzhou University, Yangzhou, 225000 Jiangsu China

**Keywords:** Hypertriglyceridemic acute pancreatitis, Recurrent acute pancreatitis, Post-acute pancreatitis diabetes mellitus

## Abstract

**Background:**

Twenty-three percent of patients are diagnosed with diabetes mellitus after the first episode of acute pancreatitis. The incidence of post-acute pancreatitis diabetes mellitus is significantly higher than that of type 1 diabetes mellitus. Some studies have concluded that the all-cause mortality and worse prognosis of diabetes after pancreatitis are higher. We predicted that number of recurrences of pancreatitis would be significantly associated with the incidences of metabolic syndrome, abdominal obesity, and post-acute pancreatitis diabetes mellitus.

**Methods:**

Patients admitted to our hospital for hypertriglyceridemic acute pancreatitis from 2013–2021 were selected for a cross-sectional study. Statistical analysis methods were used to analyze the effect of recurrences on the long-term prognosis of patients with hypertriglyceridemic acute pancreatitis.

**Results:**

In this study, 101 patients with hypertriglyceridemic acute pancreatitis were included: 60 (59.41%) in the recurrent acute pancreatitis group and 41 (40.59%) in the only one episode of acute pancreatitis group. Among all hypertriglyceridemic acute pancreatitis patients, approximately 61.4% were diagnosed with abdominal obesity, 33.7% of patients are diagnosed with metabolic syndrome, 34.7% of patients are diagnosed with diabetes mellitus, and 21.8% of patients are diagnosed with post-acute pancreatitis diabetes mellitus. Recurrent acute pancreatitis were independent risk factors for post-acute pancreatitis diabetes mellitus in patients with hypertriglyceridemic acute pancreatitis (odds ratio [OR] = 3.964, 95% confidence interval [CI] = 1.230–12.774) and the risk of post-acute pancreatitis diabetes mellitus in patients with three or more recurrent episodes was 6.607 times higher than that in patients without recurrent episodes (OR = 6.607, 95% CI = 1.412–30.916).

**Conclusions:**

Recurrence is an independent risk factor for the development of post-acute pancreatitis diabetes mellitus and is significantly associated with the number of recurrences.

**Supplementary Information:**

The online version contains supplementary material available at 10.1186/s12944-023-01840-0.

## Introduction

Acute pancreatitis (AP) is a common digestive disorder that causes local inflammation of the pancreas, with or without functional changes in other organs. The vast majority of AP is caused by gallstones (40–70%) and alcohol (25–35%). Many Chinese studies have reported that hypertriglyceridemia (HTG) has surpassed alcohol as the second leading cause of AP [1–3]. Although most patients recover completely, some patients have recurrent episodes that manifest as recurrent acute pancreatitis (RAP); a multicenter study showed that RAP occurred in approximately 29% of patients, 44.6% of whom had HTG as the etiology. Furthermore, HTG has been shown to be an independent risk factor for AP severity and recurrence [4, 5]. It has been suggested that AP heals after repeated episodes of repeated damage to the pancreatic tissue, leading to fibrosis of the necrotic area, which in turn develops into chronic pancreatitis (CP), suggesting that AP, RAP, and CP are a continuum of disease [6]. The prognosis after recurrent episodes of pancreatitis is gradually coming into the public eye, and with further research, we are gradually recognizing that some patients with AP develop sequelae, such as post-acute pancreatitis diabetes mellitus (PPDM-A), pancreatic exocrine dysfunction, and osteoporosis [7, 8].

An increasing number of studies have focused on the relationship between metabolism-related diseases and AP development; metabolic risk factors, such as obesity, hypertension, and HTG are identified as independent risk factors for the development of various AP complications. Furthermore, increased component of the metabolic syndrome results in a higher risk of more serious diseases [9]. A range of metabolic problems after AP are also receiving increasing attention, such as obesity, HTG and hyperglycemia, and pancreatic endocrine insufficiency, such as impaired glucose tolerance or diabetes mellitus. A large meta-analysis of 32 studies found that the overall prevalence of pancreatic exocrine insufficiency was 19% after mild pancreatitis and increased to 33% after severe pancreatitis [10]. Another meta-analysis of 24 prospective studies showed that 23% of the patients were diagnosed with diabetes after their first AP episode [11].

This cross-sectional study aimed to analyze the effects of recurrent episodes of hypertriglyceridemic acute pancreatitis (HTG-AP) on human metabolism and impact of the number of recurrences on long-term prognosis and found that PPDM-AP was significantly associated with HTG-AP recurrence. These findings can assist in the clinical screening of susceptible populations and allow the implementation of effective measures to stop the development of PPDM-AP and improve the quality of life of patients.

## Materials and methods

### Study population

**Fig. 1:**
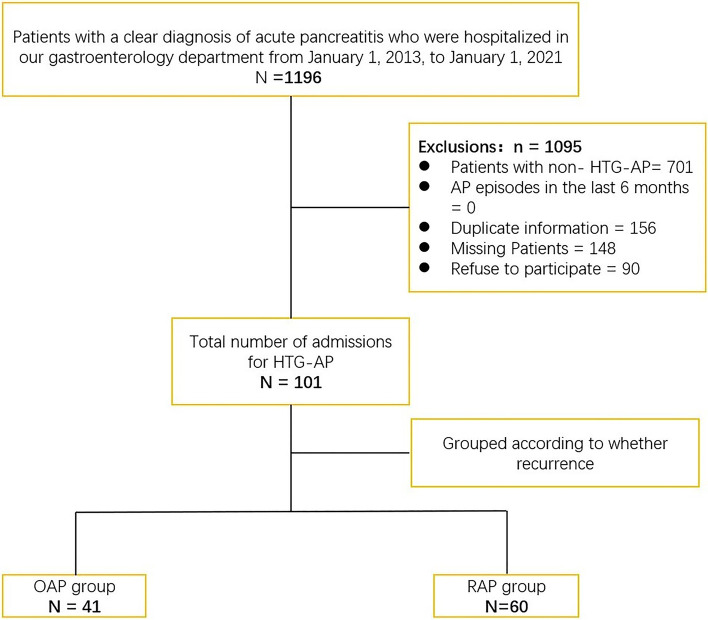
Diagram of the study flow. RAP: Recurrent acute pancreatitis. OAP: Only one episode of AP

We conducted a cross-sectional study of all patients with a clear diagnosis of HTG-AP by retrieving the clinical information of patients with AP admitted to our hospital from January 2013 to January 2021 and invited all patients who met the criteria by telephone callback to our hospital to complete questionnaires, assess liver function, renal function, routine blood and C-reactive protein, fasting and 2 h postprandial blood glucose, lipid quadruplex, and upper abdominal CT. Finally, 101 patients diagnosed with hypertriglyceridemic AP were included in this study (Fig. 1).

The inclusion Criteria were as follows:1) meeting the diagnostic criteria for AP [12]: (1) typical clinical symptoms, such as persistent abdominal pain; (2) serum amylase and/or lipase greater than three times the upper limit of normal; and (3) imaging suggesting signs of inflammation of the pancreas. 2) Etiology is clearly hypertriglyceridemia [13], That is, a serum triglyceride (TG) level greater than 11.3 mmol/L or a serum triglyceride (TG) level greater than 5.65 mmol/L in lactic acid-containing serum at the time of onset, and excluding other causes of AP. 3) Hospital admission within 72 h of illness onset.

The exclusion Criteria were as follows: (1) non-HTG-AP, (2) AP episodes in the last six months, (3) duplicate information, (4) missing patients, and (5) refusal to participate.

The study was conducted in strict compliance with the principles of the Declaration of Helsinki, informed consent was obtained from all participants, and the study was approved by the Ethics Committee of our hospital.

### Data source

All data for this study were obtained from two sources: first, current general information such as current disease, smoking, and alcohol consumption was obtained through questionnaires; hematological examinations were completed at our hospital health management center at least 6 months after discharge; and height, weight, and waist circumference were measured by doctors through professional tools at the return visit. Second, information on medical history, disease severity (Atlanta typing), and hematological results during hospitalization were obtained using the hospital information system. Ruling out transient diabetes based on Hemoglobin A1c and the use of blood glucose control medications.

### Definition

Recurrent acute pancreatitis (RAP): One or more recurrent acute pancreatitis episodes > 3 months after the first AP in complete remission [5].

One episode of acute pancreatitis (OAP): At the end of the return visit, there was only one episode of acute pancreatitis.

Abdominal obesity (AO): According to the Chinese adult AO criteria, male patients with waist circumference (WC) ≥ 90 cm or female with WC ≥ 85 cm were considered AO [14].

Metabolic syndrome (MetS) was defined as the presence of three or more of the following abnormalities: (1) central obesity: WC ≥ 90 cm in men and ≥ 85 cm in women; (2) elevated triglyceride levels ≥ 1.7 mmol/L or being treated; (3) reduced HDL-C levels < 1.0 mmol/L or specific treatment for this lipid abnormality; (4) systolic blood pressure ≥ 130 mmHg or diastolic blood pressure ≥ 85 mmHg or a clear diagnosis of hypertension; (5) fasting blood glucose level (FPG) ≥ 6.1 mmol/L or 2 h postprandial blood glucose ≥ 7.8 mmol/L or a clear diagnosis of diabetes mellitus [15].

Post-acute pancreatitis diabetes mellitus (PPDM-A): According to the 2021 American Diabetes Association (ADA) criteria, PPDM-A is defined as no prior history of diabetes prior to an AP episode and newly diagnosed diabetes after an AP [16, 17].

$$\mathrm{Waist Circumference index }(\mathrm{WTI}) =\mathrm{ WC }(\mathrm{cm}) \times \mathrm{ TG }(\mathrm{mmol}/\mathrm{L}).$$

### Statistical analysis

Statistical analyses were performed using IBM SPSS Statistics 26.0, using the mean ± standard deviation for normally distributed continuous values and the median and interquartile range (25th and 75th percentile) for non-normally distributed continuous values. Continuous values with a normal distribution in both groups were analyzed using the independent samples t-test, and continuous values with a non-normal distribution were analyzed using the two-sample rank sum test. The Kruskal–Wallis test was used to analyze continuous values of non-normal distributions for multiple groups. Count data were compared using the chi-squared test or Fisher's exact test, as appropriate. Binary logistic regression analysis was used to assess the risk factors of diabetes mellitus after pancreatitis, and hazard ratios and 95% confidence intervals (CIs) were calculated. *p* values < 0.05 were considered statistically significant.

## Results

### Clinical characteristics of the study population during hospitalization

As shown in Fig. 1, a total of 101 participants were included in this study, including 60 (59.41%) in the recurrent acute pancreatitis (RAP) group and 41 (40.59%) in the one episode of AP (OAP) group. By reviewing the general data of all participants during their first AP episode hospitalization, it was found that HTG-AP patients with a previous history of diabetes were more likely to have recurrence (Table 1), while the severity of pancreatitis during their hospitalization and inflammatory markers such as white blood cells and C-reactive protein, liver function, renal function, and lipids did not significantly affect the recurrence in HTG-AP patients.

**Table 1:** Clinical characteristics of the study population during hospitalization

	OAP (*n* = 41)	RAP (*n* = 60)	t/Z/χ^2^-value	*p*-value
Severity of first-time AP			0.876	0.740
MAP	32 (78.0%)	46 (82.1%)		
MSAP	6 (14.6%)	5 (8.9%)		
SAP	3 (7.3%)	5 (8.9%)		
Previous medical history				
Diabetes	1 (2.4%)	12 (20.0%)	6.698	0.010
Hypertension	11 (26.8%)	10 (16.7%)	1.527	0.217
Fatty liver	25 (61.0%)	40 (66.7%)	0.344	0.558
WBC (10^9^/L)	13.32 (11.06,15.08)	12.11 (10.23,15.23)	0.996	0.319
CRP (mg/L)	27.19 (10.37,122.78)	50.31 (14.29,87.06)	0.923	0.356
ALT (U/L)	38.00 (29.00,50.50)	37.00 (23.00,48.00)	0.536	0.592
AST (U/L)	28.00 (21.00,42.00)	28.00 (21.00,70.00)	0.873	0.383
GGT (U/L)	69.00 (41.00,116.50)	62.00 (43.00,109.00)	0.224	0.823
BUN (mmol/L)	4.75 (3.96,5.57)	4.81 (3.69,5.64)	0.343	0.731
UA (umol/L)	359.20 (282.50,457.70)	351.95 (278.63,450.84)	0.084	0.933
Creatinine (umol/L)	58.30 (50.00,68.00)	58.00 (47.00,71.10)	0.196	0.844
Glucose (mmol/L)	7.00 (5.96,10.31)	7.71 (5.88,11.78)	0.849	0.396
HbA1c (%)	6.60 (5.80,9.00)	7.10 (6.20,9.00)	1.019	0.308
TC (mmol/L)	6.93 (5.44,9.71)	6.62 (5.04,9.25)	1.219	0.223
TG (mmol/L)	10.31 (5.84,28.61)	10.76 (3.64,21.04)	0.561	0.575
HDL (mmol/L)	0.97 (0.70,1.27)	0.89 (0.73,1.13)	0.287	0.774
LDL (mmol/L)	1.91 (1.46,2.57)	1.66 (1.07,2.54)	0.929	0.353

*RAP* Recurrent acute pancreatitis, *OAP* One episode of AP, *MAP* Mild acute pancreatitis, *MSAP* Moderate severe acute pancreatitis, *SAP* Severe acute pancreatitis, *WBC* White blood cells, *CRP* C-reactive protein, *ALT* Alanine transaminase, *AST* Aspartate transaminase, *GGT* Gamma-glutamyltransferase, *BUN* Blood urea nitrogen, *UA* Uric acid, *HbA1c* Hemoglobin A1c, *TC* Total cholesterolemia, *TG* Total Triglyceride, *HDL* High-density lipoprotein, *LDL* Low-density lipoprotein

### Demographic and clinical characteristics of the study participants after discharge

According to the number of recurrences, patients in the RAP group were divided into one recurrence, two recurrences, and three or more recurrences (Table 2). Analysis of clinical information from patients at least 6 months after discharge revealed that WTI and glycated hemoglobin A1c levels were significantly higher in the RAP group than in the OAP group. The three or more recurrence groups had significantly higher fasting plasma glucose (FPG) and 2hPG levels than the OAP group. In the RAP group, approximately 71.7% of patients were diagnosed with abdominal obesity, 50.0% of patients were diagnosed with diabetes (all types of diabetes), and 48.33% of patients were diagnosed with metabolic syndrome, 50% of patients required lipid control with medication. Among those with three or more recurrences, up to 75.0% were diagnosed with diabetes and 75.0% were diagnosed with metabolic syndrome, 83.3% of patients required lipid control with medication. By analyzing the use of lipid-lowering medications in HTG-AP patients, we also found fibrates to be the medications of choice (An additional file shows this in more detail (see Additional file 1: Figure S1)). Notably, approximately 9.8% of the population in the OAP group and approximately 30.0% of the population in the RAP group subsequently developed PPDM-A. Among all recurrences, approximately 38.9% of the population with two recurrences subsequently developed PPDM-A, and approximately 41.7% of the population with three or more recurrences subsequently developed PPDM-A, both of which were significantly higher than the probability of PPDM-A in the OAP group. From the above study, it was found that multiple episodes of HTG-AP may lead to metabolic disorders in patients, such as recurrent episodes of HTG-AP leading to higher WTI, and are more likely to develop AO and PPDM-A, especially with adverse effects on glycemic control, with significantly higher FPG, 2hPG, and glycated hemoglobin levels in patients with three or more recurrences.

**Table 2:** Demographic and clinical characteristics of the Study Participants after discharge

	OAP (*n* = 41)	RAP (*n* = 60)	Recurrence frequency		
			1 recurrence (n = 30)	2 recurrences (n = 18)	≥ 3 recurrence (n = 12)
Sex					
Male	32 (78.0%)	45 (75%)	24 (80.0%)	14 (77.8%)	7 (58.3%)
Female	9 (22.0%)	15 (25%)	6 (20.0%)	4 (22.2%)	5 (41.7%)
Age (years)	45.00 (38.50, 51.50)	42.00 (36.00,52.00)	43.50 (36.75,53.25)	38.50 (32.75,53.25)	44.00 (38.25,53.75)
BMI	26.15 (24.33,27.52)	26.20 (24.28,28.33)	26.02 (24.35,27.73)	26.98 (24.88,28.52)	26.00 (23.00,26.00)
AO	19 (46.3%)	43 (71.7%)^a^	19 (63.3%)	15 (83.3%)^a^	9 (75.0%)
WTI	265.60 (156.07,657.76)	420.34 (213.60,1048.48)^a^	357.55 (172.27,720.78)	515.32 (270.39,1058.11)	867.48 (311.09,1349.43)
Smoking	23 (56.1%)	38 (63.3%)	21 (70.0%)	10 (55.6%)	7 (58.3%)
Alcohol	27 (65.8%)	39 (65.0%)	22 (73.3%)	10 (55.6%)	7 (58.3%)
Lipid-lowering medications	12 (29.3%)	30 (50.0%)	12 (40.0%)	8 (44.4%)	10 (83.3%)^a b c^
Comorbidities (n,%)					
Diabetes	5 (12.2%)	30 (50.0%)^a^	12 (40.0%)^a^	9 (50.0%)^a^	9 (75.0%)^a b^
PPDM-A	4 (9.8%)	18 (30.0%)^a^	6 (20.0%)	7 (38.9%)^a^	5 (41.7%)^a^
Hypertension	12 (29.3%)	15 (15.00%)	8 (26.7%)	3 (16.7%)	4 (33.3%)
New onset of Hypertension	5 (12.2%)	7 (11.7%)	3 (10.0%)	2 (11.1%)	2 (16.7%)
Fatty liver	14 (34.1%)	28 (46.7%)	9 (30.0%)	12 (66.7%)^a b^	7 (58.3%)
New onset of Fatty liver	3 (7.3%)	6 (10.0%)	1 (3.3%)	4 (22.2%)	1 (8.3%)
Mets	5 (12.2%)	29 (48.3%)^a^	10 (33.3%)^a^	10 (55.6%)^a^	9 (75.0%)^a b^
WBC (109/L)	6.28 ± 1.46	7.01 ± 3.59	6.16 (5.15,8.18)	6.96 (5.36,8.36)	5.89 (5.48,6.96)
CRP (mg/L)	1.14 (0.47,2.36)	1.15 (0.53,2.53)	1.14 (0.43,2.38)	1.67 (0.92,3.92)	1.00 (0.32,1.60)
Total bilirubin (umol/L)	13.20 (7.60,13.20)	11.80 (9.30,16.15)	12.10 (9.60,17.05)	12.60 (10.55,17.47)	9.65 (8.92,13.20)
Direct bilirubin (umol/L)	3.30 (2.75,4.00)	3.00 (2.40,3.68)	3.15 (2.37,4.15)	3.05 (2.65,3.40)	2.45 (1.75,2.97)
Indirect bilirubin (umol/L)	9.85 (6.03,13.25)	8.80 (6.73,11.25)	8.80 (6.65,11.47)	9.55 (7.72,13.85)	7.75 (6.07,10.25)
ALT (U/L)	28.20 (18.35,48.00)	28.85 (20.00, 40.75)	29.85 (19.25,82.80)	30.00 (19.25,45.00)	27.50 (12.50,35.75)
AST (U/L)	25.20 (19.35,31.00)	22.10 (18.85,31.43)	22.40 (18.77,33.30)	22.80 (19.55,28.87)	20.50 (17.75,26.67)
GGT (U/L)	42.10 (24.55,83.25)	40.85 (22.95,68.37)	55.45 (26.42,90.37)	37.25 (19.32,52.47)	37.10 (21.15,67.25)
FPG (mmol/L)	6.25 (5.31,8.91)	6.80 (5.62,10.00)	6.44 (5.75,7.98)	6.25 (5.34,12.14)	10.14 (7.40,12.74)^a b^
2hPG (mmol/L)	8.36 (6.27,14.26)	10.89 (7.41,14.97)	9.79 (7.01,11.71)	8.37 (6.39,16.95)	14.85 (12.31,18.46)^a b^
HbA1c (%)	5.50 (5.25,7.00)	6.00 (5.40,7.80)^a^	5.75 (5.40,7.12)	6.00 (5.15,9.30)	7.90 (6.75,9.27)^a b^
BUN (mmol/L)	5.38 (4.49,6.22)	5.58 (4.76,6.59)	5.63 (4.98,6.65)	5.40 (4.20,6.62)	5.54 (4.41,6.27)
UA (umol/L)	333.60 (281.60,428.50)	346.15 (288.80,405.82)	369.50 (298.70,484.20)	349.55 (306.05,405.67)	311.15 (287.35,361.00)
Creatinine (umol/L)	68.10 (59.00, 79.10)	67.35 (53.70,79.95)	68.85 (55.60,87.55)	58.80 (50.07,71.30)	63.70 (33.65,84.00)
TG (mmol/L)	3.00 (1.76,7.80)	4.50 (2.64,9.58)	3.94 (1.94,8.01)	5.02 (3.10,10.19)	8.62 (3.70,14.91)
TC (mmol/L)	5.20 (4.51,5.75)	5.58 (4.42,6.49)	5.38 (4.38,5.99)	5.74 (4.75,6.60)	5.88 (4.16,7.87)
HDL (mmol/L)	1.04 (0.91,1.15)	0.96 (0.85,1.16)	0.98 (0.86,1.22)	0.94 (0.82,1.11)	0.91 (0.84,1.01)
LDL (mmol/L)	2.3 (1.87,2.96)	2.04 (1.53,2.68)	2.31 (1.66,3.11)	2.34 (1.68,2.80)	1.53 (1.36, 1.76)

*RAP* Recurrent acute pancreatitis, *OAP* One episode of AP, *BMI* Body mass index, *AO* Abdominal obesity, *WTI* Waist circumference index, *PPDM-A* Post-acute pancreatitis diabetes mellitus, *Mets* Metabolic syndrome, *WBC* White blood cells, *CRP* C-reactive protein, *MCHC* Mean corpuscular hemoglobin contentration, *RDW* Red cell distribution width, *RBC* Red blood cells, *ALT* Alanine transaminase, *AST* Aspartate transaminase, *GGT* Gamma-glutamyltransferase, *FPG* Fasting plasma glucose, *2hPG* 2 h postprandial blood glucose, *HbA1c* Hemoglobin A1c, *BUN* Blood urea nitrogen, *UA* Uric acid, *TG* Total Triglyceride, *TC* Total cholesterolemia, *HDL* High-density lipoprotein, *LDL* Low-density lipoprotein, *GGT* Gamma-galactosyltransferase, *LDH* Lactate de-hydrogenase, *OR* Odds ratio

^a^* P* < 0.05, Compared with the OAP group

^b^* P* < 0.05, Compared with the 1 recurrence group

^c^* P* < 0.05, Compared with the 2 recurrence group

### Diet component detail analysis

**Fig. 2:**
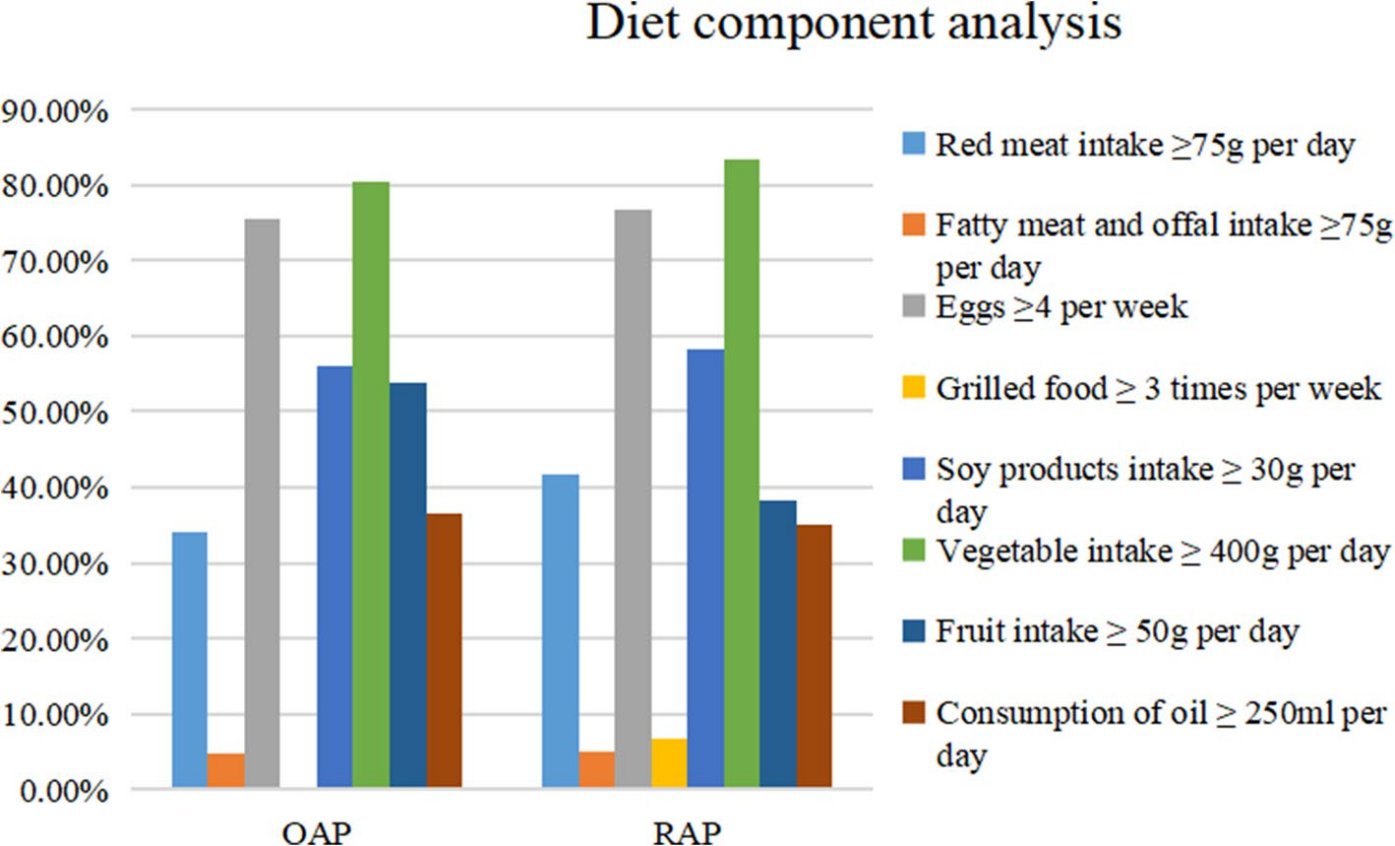
Diet component analysis

The questionnaire was completed anonymously by respondents under the guidance of a uniformly trained surveyor using a one-person, one-form questionnaire method that included dietary components. As shown in Fig. 2, there were no significant differences (*P* > 0.05) in the intake of meat, eggs, fried foods, soy products, vegetables, or edible oils between the two groups. Overall, both groups consumed predominantly red meat, more than 70% consumed ≥ 4 eggs per week, more than 50% consumed ≥ 30 g of soy products per day, more than 80% consumed ≥ 400 g of vegetables per day, about 40% consumed ≥ 50 g of fruit per day, and more than 30% consumed greater than or equal to 250 ml of cooking oil per day. This study found no significant differences in the postdischarge dietary structure of patients with HTG-AP.

### Analysis of the risk of PPDM-A

**Fig. 3:**
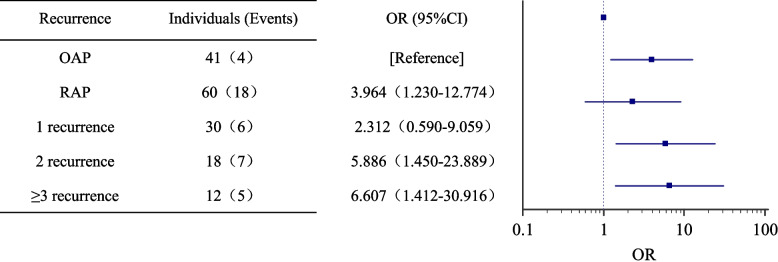
Analysis of the number of AP recurrences on the risk of PPDM-A

In the above study, it was found that among the 101 patients with HTG-AP, 22 (21.9%) developed PPDM-A after an AP episode. Therefore, we further analyzed the risk factors for PPDM-A using univariate logistic regression (Table 3) and found that hypertension, fatty liver, and AO were not significantly associated with PPDM-A development, whereas AP recurrence was significantly associated with PPDM-A (OR:3.964,95% CI:1.230–12.774). Next, the effect of the number of AP recurrences on PPDM-A was further analyzed (Fig. 3), and it was found that the OR of PPDM-A in the group with 2 recurrences was 5.886, (95% CI:1.450–23.886), and the OR of PPDM-A in the group with 3 or more recurrences was 6.607 (95% CI:1.412- 30.916).

**Table 3:** Univariate logistic regression analysis for factors associated with PPDM-A

Variables	*Adjusted p*- Value	Adjusted OR (95% Confidence Interval)
Hypertension	0.252	1.805 (0.657–4.956)
Fatty liver	0.575	0.756 (0.285–2.007)
AO	0.461	1.459 (0.535–3.979)
RAP	0.021	3.964 (1.230–12.774)

## Discussion

In our study, of all 1196 AP patients admitted to our hospital in the last 8 years, HTG-AP accounted for 41.39%. The recurrence rate of HTG-AP in this study was as 59.41%, which is much higher than reported before. This may be due to the fact that patients with recurrent episodes preferred to be followed up. Due to the high prevalence and recurrency of HTG-AP in our center, we paid more attention to HTG-AP patients.

In addition, we collected information on lipid-lowering medications for all participants (An additional file shows this in more detail (see Additional file 1: Figure S1)). Among all patients with HTG-AP, 51.48% (52/101) required lipid-lowering drug interventions. Specifically, 32.67% (33/101) used fibrates.

We found that among all HTG-AP patients, 61.4% (62/101) were diagnosed with AO, 33.7% (34/101) with MetS, 34.7% (35/101) with diabetes, and 21.8% (22/101) with PPDM-A. The data were 71.7% (43/60), 48.3% (29/60), 50.0% (30/60), and 30.0% (18/60) in recurrent HTG-AP patients, especially. This further indicates the important role of metabolism-related diseases in the development and recurrency of AP. Similary to our study, majority of HTG-AP patients had metabolic disorders such as diabetes mellitus, hypertension and obesity [18]. Hypertriglyceridemia and diabetes were even independent risk factors for AP recurrence [19].

However, there are few studies related to the effect of AP recurrence on human metabolism, especially PPDM-A, which was closely related to impaired pancreatic endocrine function after pancreatitis. All-cause mortality in PPDM-A is 13% higher than that in type 2 diabetes, with nearly 15 excess deaths per 1000 person-years [20, 21]. Thus, our research further concerned about the influence of recurrency of HTG-AP to PPDM-A.

In this study, our data showed that the prevalence of PPDM-A was 21.8% in patients with HTG-AP and that hypertension, fatty liver, and AO were not significantly associated with PPDM-A. Recurrent episodes were an independent risk factor for PPDM-A in patients with HTG-AP and were significantly associated with the number of AP recurrences, with the risk of PPDM-A in patients with three or more recurrences being 6.607 times higher than that in patients with only one episode of AP. In line with our findings, a multicenter retrospective cohort study in 2022 in China that included 6009 participants demonstrated and quantified for the first time the prevalence of PPDM-A after the first episode of AP at 6.2%; patients with PPDM-A were more likely to have HTG-AP, and stress hyperglycemia, hyperlipidemia, nonalcoholic fatty liver disease (NAFLD), and recurrent AP were found by multifactorial analysis to be independent risk factors for PPDM-A [22]. A recent study found that 8.8% of 329 patients with AP were diagnosed with PPDM-A, of which 6.37% were diagnosed within one month of acute onset and 2.42% after one month, and obesity was an independent risk factor for PPDM-A [23]. Approximately 60% of children diagnosed with PPDM-A have RAP, and hypertriglyceridemia is a risk factor for PPDM-A [24]. We believe that patients with recurrences are more likely to have metabolism-related diseases than those without recurrences. To the best of our knowledge, this is the first study to analyze the effect of the number of recurrences on PPDM-A scores in a population with a high HTG-AP rate.

Also, in our results, patients with recurrent HTG-AP have more difficult glycemic control, and this may correlate with the number of relapses: when the number of relapses exceeds three, their fasting blood glucose is approximately 10.41 mmol/L, 2 h postprandial blood glucose is approximately 14.85 mmol/L, and glycosylated hemoglobin is approximately 7.90%. HTG-AP patients with more than three recurrences experience significant uncontrollable hyperglycemia. One large retrospective cohort study in 2017 that included 31,789 adults with diabetes found that the incidence of PPDM-A was significantly higher than that of type 1 diabetes, at 2.59 cases per 100,000 person-years and 1.8% of Adult-Onset Diabetes, and that patients with PPDM-A had more difficult glycemic control and greater insulin requirements [25]. This further proved that the recurrency of HTG-AP would impair the pancreatic endocrine function. In 2018, a study that first assessed the relationship between AO and insulin resistance (IR) in the post-pancreatitis setting found significantly higher IR in patients with AO [26], insulin resistance is considered to be the underlying pathophysiological phenomenon of various chronic metabolic disorders such as diabetes, obesity, and metabolic syndrome. However, a larger sample size study is needed to confirm this.

AP is the most common etiology of PPDM-A, the underlying mechanism of which has not yet been clearly established. Transient or persistent pancreatic injury may induce pancreatic autoimmune inflammation, and inflammation in the surrounding exocrine tissue may extend to nearby pancreatic islets, leading to an immune response against islet antigens [27]. Existing studies suggest that pancreatic injury decreases the number of islets, which is an important cause of diabetes after severe necrotizing AP. Lipolysis underlies the pathogenesis of PPDM-A that occurs in patients with mild AP. After pancreatitis, lipolysis increases in patients with diabetes by increasing the levels of glycerol and triglycerides, thereby lowering blood glucose levels. Increased levels of glycerol increase IL-6 and β-hydroxybutyrate, and β-hydroxybutyrate lowers blood glucose, while The inflammatory factor IL-6 leads to impaired phosphorylation of the insulin receptor and insulin receptor substrate-1, resulting in insulin resistance (a 1 ng/ml increase in IL-6 is associated with a 0.7% increase in insulin resistance) [28, 29]. In 2020, proper exercise was suggested to reverse the increase in diabetes-related hormones after pancreatic inflammation and possibly prevent the development of PPDM-A [29]. Further research is needed to explore the mechanisms that identify targets and increase the possibility of reducing the burden of AP through tertiary prevention measures.

Compared with type 2 diabetes, PPDM-A has a higher mortality rate and a greater risk of cancer [30]. Herein, we found that recurrent AP is significantly associated with PPDM-A. Therefore, we recommend that patients with recurrent AP undergo a dynamic review of glycosylated hemoglobin, fasting glucose, and 2hPG. Moreover, joint multidisciplinary participation is necessary to customize disease management strategies based on patient characteristics to prevent early disease progression. Notably, the optimal time to review glycosylated hemoglobin, FPG, and 2hPG is at least 3 months after discharge from the hospital to minimize discrepancies [31]. In terms of treatment, early treatment with biguanides with antitumor effects is beneficial. Additionally, it is important to avoid hypoglycemic drugs that increase the risk of hypoglycemia, such as sulfonylureas, due to the high blood glucose fluctuations seen in PPDM-A [32–34].

### Limitations

There are limitations to this study. First, it was a single-center study, which makes it difficult to recruit a sufficient number of representative subjects. Through Power analysis (Supplemental Figs. 2 and 3), we determined that AP recurrence could significantly contribute to the development of PPDM-A. A multicenter study with a larger sample size is necessary to further explore the relationship between the number of AP recurrences and PPDM-A in greater detail (An additional file shows this in more detail (see Additional file 2: Figures S2 and Additional file 3: Figures S3)). Second, regarding the diagnosis of PPDM-A, we only reviewed the medical history of all participants based on whether they had newly developed diabetes after the disease [16], and some patients lacked data such as blood glucose and glycated hemoglobin, which can lead to false-negative results. Thirdly As shown in Figure S4 (An additional file shows this in more detail (see Additional file 4: Figure S4)), the population showed a trend of progressive increase in the number of AP episodes with increasing years of follow-up; therefore, we cannot ignore the fact that HTG patients are more likely to experience recurrence and that there is a certain time effect. Follow-up studies should be designed to investigate this issue further. Familial chylomicronemia syndrome (FCS) poses a higher risk of AP compared to other types of hypertriglyceridemia, with approximately 60% to 80% of patients with FCS experiencing at least one episode of AP during their lifetime [35]. Familial chylomicronemia syndrome (FCS) poses a higher risk of AP compared to other types of hypertriglyceridemia, with approximately 60% to 80% of patients with FCS experiencing at least one episode of AP during their lifetime [35, 36]. Given the low prevalence of FCS and the high reliance on genetic sequencing for diagnosis, we did not strictly differentiate between types of hypertriglyceridemia, which is another limitation of this study.

## Conclusions

In conclusion, this study investigated the effect of recurrence on human metabolism-related diseases by analyzing the general data of the HTG-AP, RAP, and OAP groups, and found that recurrent episodes of HTG-AP lead to uncontrollable blood glucose levels and are an independent risk factor for PPDM-A, which is significantly associated with the number of pancreatitis episodes.

## Supplementary Information

**Additional file 1:** **Figure S1.** Survey of lipid-lowering medication.

**Additional file 2:** **Figure S2.** Power analysis of the relationship between AP recurrence and PPDM-A.

**Additional file 3:** **Figure S3.** Power analysis of the relationship between the number of AP recurrences and PPDM-A.

**Additional file 4:** **Figure S4.** Analysis of the relationship between the number of acute pancreatitis episodes and the follow-up time.

## Data Availability

All data are contained in the article. The raw data will be shared upon request: Contact corresponding author.

## Abbreviations

RAP: Recurrent acute pancreatitis
OAP: One episode of AP
HTG: Hypertriglyceridemia
PPDM-A: Post-acute pancreatitis diabetes mellitus
HTG-AP: Hypertriglyceridemic acute pancreatitis
MAP: Mild acute pancreatitis
MSAP: Moderate severe acute pancreatitis
SAP: Severe acute pancreatitis
BMI: Body mass index
AO: Abdominal obesity
WTI: Waist circumference index
WC: Waist circumference
Mets: Metabolic syndrome
WBC: White blood cells
CRP: C-reactive protein
MCHC: Mean corpuscular hemoglobin contentration
RDW: Red cell distribution width
RBC: Red blood cells
ALT: Alanine transaminase
AST: Aspartate transaminase
GGT: Gamma-glutamyltransferase
FPG: Fasting plasma glucose
2hPG: 2 Hours postprandial blood glucose
HbA1c: Hemoglobin A1c
BUN: Blood urea nitrogen
UA: Uric acid
TG: Total Triglyceride
TC: Total cholesterolemia
HDL: High-density lipoprotein
LDL: Low-density lipoprotein
GGT: Gamma-galactosyltransferase
LDH: Lactate de-hydrogenase
OR: Odds ratio
