# Supplementary figures and images for: Number of recurrences is significantly associated with the post-acute pancreatitis diabetes mellitus in a population with hypertriglyceridemic acute pancreatitis
Source: Lipids Health Dis. 2023 Jun 29;22:82. doi: 10.1186/s12944-023-01840-0 (PMC10308690; doi:10.1186/s12944-023-01840-0)

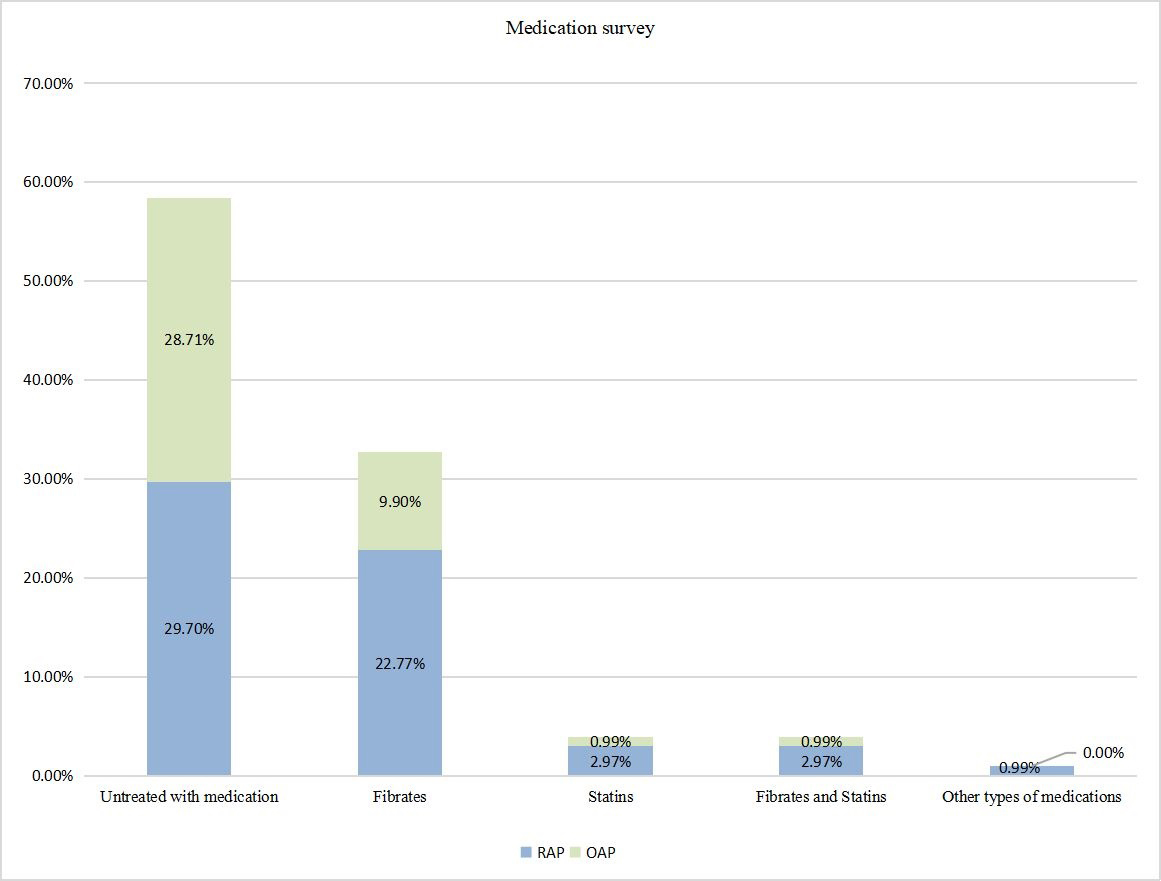

Supplement: Supplementary file 1 — Additional file 1: Figure S1. Survey of lipid-lowering medication. [file 12944_2023_1840_MOESM1_ESM.jpg]

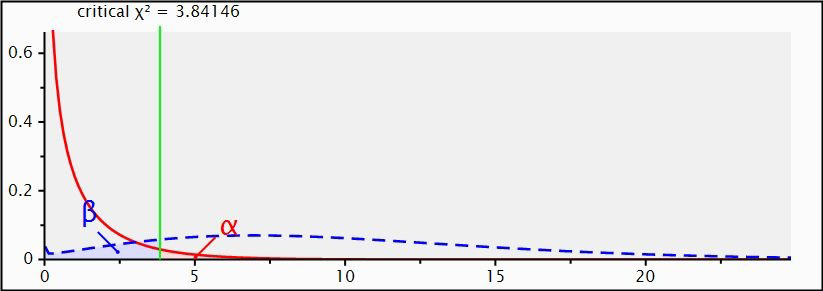

Supplement: Supplementary file 2 — Additional file 2: Figure S2. Power analysis of the relationship between AP recurrence and PPDM-A. [file 12944_2023_1840_MOESM2_ESM.jpg]

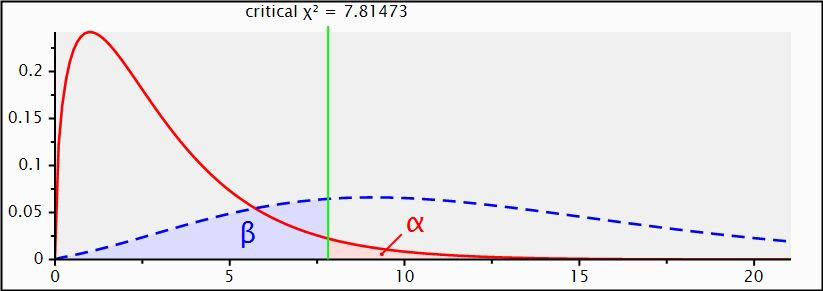

Supplement: Supplementary file 3 — Additional file 3: Figure S3. Power analysis of the relationship between the number of AP recurrences and PPDM-A. [file 12944_2023_1840_MOESM3_ESM.jpg]

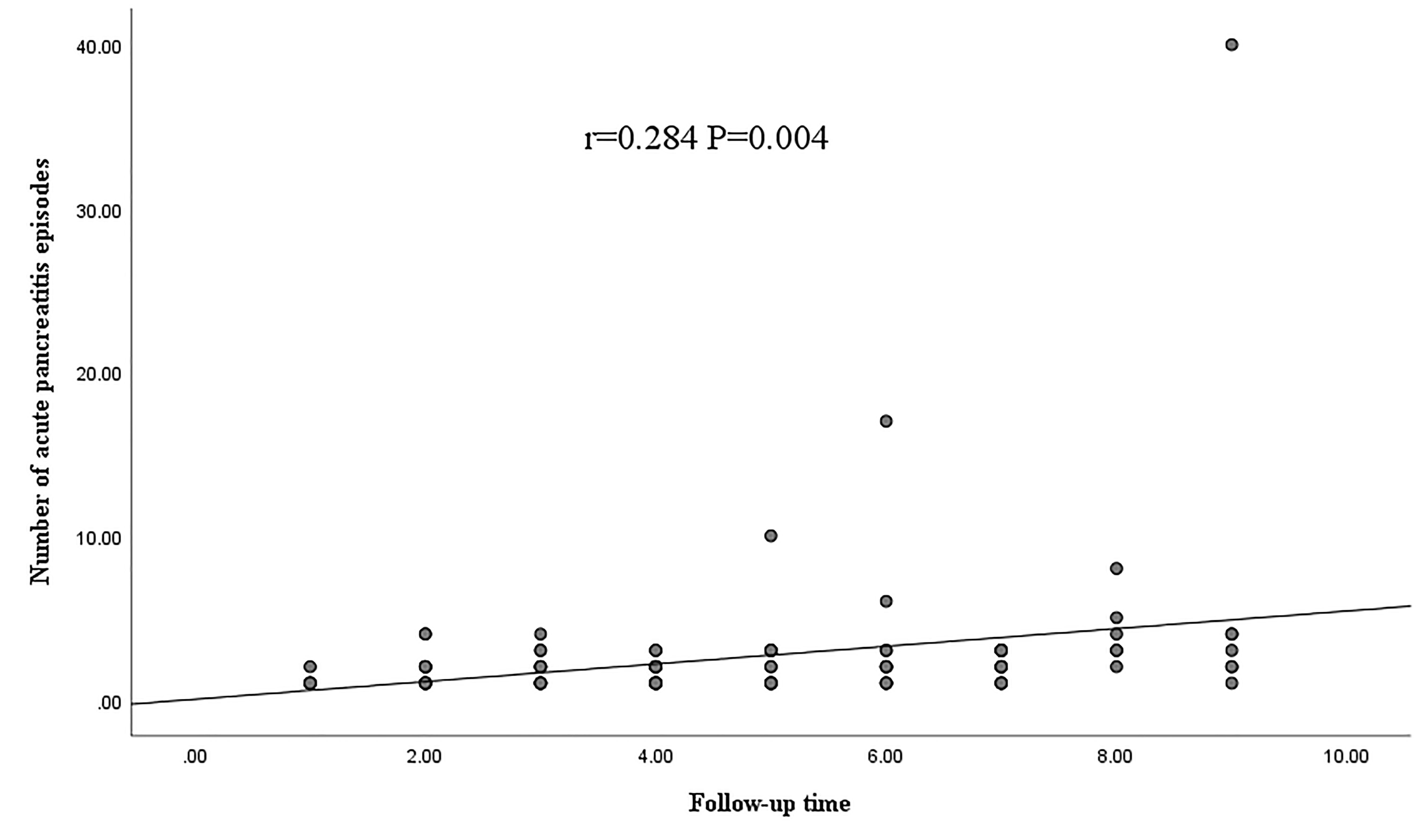

Supplement: Supplementary file 4 — Additional file 4: Figure S4. Analysis of the relationship between the number of acute pancreatitis episodes and the follow-up time. [file 12944_2023_1840_MOESM4_ESM.jpg]
